# Instability of Liquid Film with Odd Viscosity over a Non-Uniformly Heated and Corrugated Substrate

**DOI:** 10.3390/nano13192660

**Published:** 2023-09-28

**Authors:** Danting Xue, Ruigang Zhang, Quansheng Liu, Zhaodong Ding

**Affiliations:** School of Mathematical Science, Inner Mongolia University, Hohhot 010021, China

**Keywords:** liquid film, odd viscosity, Marangoni effect, bottom steepness, instability

## Abstract

The effect of odd viscosity on the instability of liquid film along a wavy inclined bottom with linear temperature variation is investigated. By utilizing the long-wave approximation, the non-linear evolution equation of the free surface is derived. By applying the normal mode method, the linear instability of thin film flow is investigated. With the help of multi-scale analysis methods, the weakly non-linear instability of thin film flow is also investigated. The results reveal that the Marangoni effect caused by non-uniform temperature distribution promotes the instability of the liquid film, while the odd viscosity has a stabilizing effect. In addition, for a positive local inclination angle θ, an increase in bottom steepness ζ inhibits the instability of the liquid film flow. In contrast, with a negative local inclination angle θ, increased bottom steepness ζ promotes the instability of the liquid film flow. The results of the temporal linear instability analysis and the weakly non-linear instability analysis have been substantiated through numerical simulations of the non-linear evolution equations.

## 1. Introduction

Liquid membranes find extensive applications in various industrial engineering fields, including thin film evaporators and liquid film dust collectors used in chemical equipment, as well as thin liquid film cooling of large-scale integrated circuits [1,2]. Additionally, the instability analysis of liquid films flowing along inclined or vertical planes is of significant relevance in the coating industry, where it plays a crucial role in microchip manufacturing, paper coating, magnetic film coating, and other related processes [3,4,5,6]. The complexities of liquid film flow have therefore garnered significant attention from researchers and practitioners alike, making it an important and constantly studied area of interest.

Benjamin [7] and Yih [8] were among the pioneers in studying the stability of liquid film flow over inclined planes. They focused on solving the linear stability problem for the fundamental flow of constant thickness and determined the critical Reynolds number $Re_{c}$. When the Reynolds number exceeds $Re_{c}$, the flow of liquid film becomes unstable. Samanta [9,10] conducted an analysis of the linear stability of thin liquid film flow on a non-uniformly heated inclined plane, neglecting the effect of inertial forces and employing the canonical mode method. Simultaneously, the long wave perturbation method was utilized to solve the control equation, leading to the derivation of a nonlinear surface wave equation. This enabled the analysis of linear, nonlinear, and sideband stability of liquid film flow on a vertically non-uniformly heated substrate. For additional studies on the stability of a thin film flow over the beveled and vertical planes, reference can be made to articles by Bauer and Kerczek [11], Hanratty [12] and Craster and Matar [13], among others. As for the stability of the film flow on a heated or inclined plane, research can be found in the work of Kalliadasis et al. [14], Sadiq et al. [15] and Mukhopadhyay et al. [16]. Gjevik [17] and Nakaya [18] extended the stability investigation of thin films to consider nonlinear stability.

Nevertheless, the aforementioned studies have primarily focused on liquid film flows over inclined or vertical substrates. In industrial equipment or other practical applications, certain substrates unavoidably have uneven surfaces. Solely investigating plane slopes may introduce bias when applied to non-level cases. In other words, the substrate’s structural characteristics significantly influence the stability of liquid film flow. The fluid dynamics of falling liquid films over undulating surfaces has attracted considerable attention, with early work by Pozrikidis [19] examining free surface Stokes flow along a sinusoidal base. Bielarz [20] discussed the stability of thin free surface liquid films flowing over structural surfaces, employing both one-dimensional and two-dimensional calculations. Wierschem and Aksel [21] performed a linear stability analysis of a Newtonian liquid film flowing down an inclined wavy plane. Their study investigated how wavy bottom variations, significantly longer than the film thickness, impact the stability of steady film flow compared to that over a flat inclined plane. Trifonov [22] studied the flow of a viscous liquid film along an inclined corrugated surface, using an integral model and employing Floquet theory to analyze the stability of nonlinear steady-state flows under various conditions. For research on the influence of substrate structure on film flow, references can be made to the works of Heining and Aksel [23], as well as Tougou [24]. These studies shed light on the crucial role played by the substrate’s unevenness or corrugations in determining the stability characteristics of a liquid film flow.

In recent years, odd viscosity has emerged as a prominent subject of investigation in the study of thin film flow stability. Odd viscosity [25,26] is the non-dissipative component of the viscosity tensor and is contained in its antisymmetric part. Avron [27,28] made a break-through discovery by demonstrating that in a classical fluid, when time-reversal symmetries are broken, either spontaneously or due to an external magnetic field or rotation, the viscosity tensor can have a non-zero odd part that gives rise to a dissipationless linear response coefficient known as odd or Hall viscosity. In natural situations where the time-reversal symmetry of a classical liquid is broken, odd viscosity effects are commonly observed in biological [29], granular [30] and colloidal [31] systems. Kirkinis and Andreev [32] explored the impact of odd viscosity on the thermocapillary instability of a viscous liquid film flowing along a uniformly heated solid substrate, while considering a fixed temperature gradient across the free surface. Their findings revealed that the initial wave of odd viscosity can suppress thermocapillary instability, leading to enhanced stability of the thin liquid film. A comprehensive exploration of odd viscosity in fluid dynamics was presented by Lapa et al. [33], providing a broader understanding of this intriguing phenomenon. Additionally, Zhao and Jian [34] investigated the effect of odd viscosity on the stability of a falling thin film in the presence of an electromagnetic field. Employing the lubrication approximation, they derived a new nonlinear evolution equation for the free surface that takes into account the influence of odd viscosity. Through linear and weakly nonlinear stability analyses, they found that odd viscosity has a stabilizing effect on the system. These studies collectively contribute to our understanding of the significant role that odd viscosity plays in influencing the stability characteristics of thin film flows, and highlight its relevance in various physical systems and applications.

This paper is centered around investigating the influence of odd viscosity on the instability of liquid film flowing down an undulated inclined plate with linear temperature variation. Specifically, the study examines the effects of odd viscosity, thermocapillary effects and bottom steepness on flow instability, leading to corresponding conclusions.

## 2. Mathematical Model

**Figure 1:**
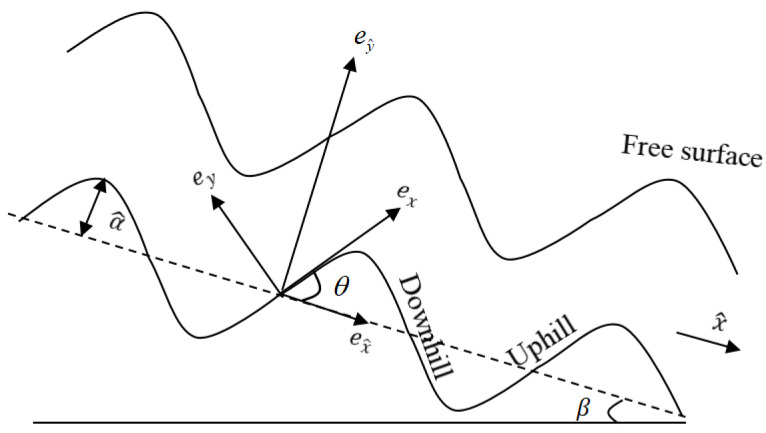
The schematic diagram of the physical model.

We consider the flow of an incompressible viscous Newtonian fluid driven by gravity in two dimensions. The fluid flows down an inclined, corrugated substrate with uneven heating, as depicted in Figure 1. The Cartesian coordinate system $e_{\hat{x}}$, $e_{\hat{y}}$ has an angle β with respect to the horizontal, and the base contour $\hat{b}\left(\hat{x}\right)$ is periodic with amplitude $\hat{a}$ and wavelength $\hat{\lambda }$, where $\hat{x}$ is in the direction of the main flow. The substrate profile is undulated, so a local curvilinear coordinate system is introduced, and the when thickness of the thin films are thin enough compared to the curvature radius of the bottom, the flow (u,v) is still mainly parallel to the bottom [35]. Therefore, at every point of the bottom, a local coordinate system $e_{x}$, $e_{y}$ is defined, where $e_{x}$ is the tangent and $e_{y}$ is normal to the base. For any point *P* in the fluid, the coordinates of the curve are the arc length *x* of the base and the distance *y* along the $e_{y}$ to the base. In $e_{\hat{x}}$, $e_{\hat{y}}$ coordinates $P=(\hat{x}-\sin\theta y,\hat{b}\left(\hat{x}\right)+\cos\theta y)$, where $\theta =\theta \left(\hat{x}\right)=\arctan\left(\partial \hat{b}\left(\hat{x}\right)/\partial \hat{x}\right)$ is the local inclination angle between $e_{\hat{x}}$ and $e_{x}$.

This is the considered film flows along a moderate steepness undulating substrate and has a long length compared to the film thickness. The substrate curvature κ is denoted by

(1) $$\kappa \left(\hat{x}\right)=-\frac{\partial^{2}\hat{b}\left(\hat{x}\right)}{\partial {\hat{x}}^{2}}{\left[1+{\left(\frac{\partial \hat{b}\left(\hat{x}\right)}{\partial \hat{x}}\right)}^{2}\right]}^{-3/2}.$$

For further details on the transformation to curvilinear coordinates, we refer to the study of Wierschem et al. [36].

We assume that such fluids are time reversal symmetry breaking, and the falling film problem should consider both even $\tau^{e}$ and odd $\tau^{o}$ viscosity coefficients. In this case, the Cauchy stress tensor τ is expressed as

(2) $$\tau =\tau^{e}+\tau^{o},$$

(3) $$\tau_{ij}^{e}=-p\delta_{ij}+\eta^{e}\left(\frac{\partial u_{i}}{\partial x_{j}}+\frac{\partial u_{j}}{\partial x_{i}}\right),$$

(4) $$\begin{array}{cc} \tau_{ij}^{o}= & -\eta^{o}\left(\delta_{i1}\delta_{j1}-\delta_{i2}\delta_{j2}\right)\left(\frac{\partial u_{1}}{\partial x_{2}}+\frac{\partial u_{2}}{\partial x_{1}}\right)+\eta^{o}\left(\delta_{i1}\delta_{j2}-\delta_{i2}\delta_{j1}\right)\left(\frac{\partial u_{1}}{\partial x_{1}}-\frac{\partial u_{2}}{\partial x_{2}}\right), \end{array}$$

where *i*, *j* = 1, 2, $\eta^{e}$ and $\eta^{o}$ denote the odd and even viscosity coefficients, respectively.

The dynamic properties of an incompressible fluid can be described by the continuity equation and the momentum equation. Referring to Wierschem et al. [36], the following equations are derived based on the curvilinear coordinate transformation considering the odd viscosity and the Marangoni effect. And, to simplify the notations, we set $u=u_{1},\;v=u_{2},$ $x=x_{1},$ and $y=x_{2}$.

(5) $$\frac{1}{1+\kappa y}\left(\frac{\partial u}{\partial x}+\kappa v\right)+\frac{\partial v}{\partial y}=0,$$

(6) $$\begin{array}{cc} \; & \rho \left(\frac{\partial u}{\partial t}+\frac{1}{1+\kappa y}u\left(\frac{\partial u}{\partial x}+\kappa v\right)+v\frac{\partial u}{\partial y}\right)=-\frac{1}{1+\kappa y}\frac{\partial p}{\partial x}+\rho g\sin\left(\beta -\theta \right)\\ \; & +\eta^{e}\left[{\left(\frac{1}{1+\kappa y}\right)}^{3}\frac{\partial \kappa }{\partial x}\left(v-y\frac{\partial u}{\partial x}\right)+{\left(\frac{1}{1+\kappa y}\right)}^{2}\left(\frac{\partial^{2}u}{\partial x^{2}}-\kappa^{2}u+2\kappa \frac{\partial v}{\partial x}\right)+\frac{1}{1+\kappa y}\kappa \frac{\partial u}{\partial y}+\frac{\partial^{2}u}{\partial y^{2}}\right]\\ \; & -\eta^{o}\left[-{\left(\frac{1}{1+\kappa y}\right)}^{3}\frac{\partial \kappa }{\partial x}\left(u+y\frac{\partial v}{\partial x}\right)+{\left(\frac{1}{1+\kappa y}\right)}^{2}\left(\frac{\partial^{2}v}{\partial x^{2}}-\kappa^{2}v-2\kappa \frac{\partial u}{\partial x}\right)+\frac{1}{1+\kappa y}\kappa \frac{\partial v}{\partial y}+\frac{\partial^{2}v}{\partial y^{2}}\right], \end{array}$$

(7) $$\begin{array}{cc} \; & \rho \left(\frac{\partial v}{\partial t}+\frac{1}{1+\kappa y}u\left(\frac{\partial v}{\partial x}-\kappa u\right)+v\frac{\partial v}{\partial y}\right)=-\frac{\partial p}{\partial y}-\rho g\cos\left(\beta -\theta \right)\\ \; & +\eta^{e}\left[-{\left(\frac{1}{1+\kappa y}\right)}^{3}\frac{\partial \kappa }{\partial x}\left(u+y\frac{\partial v}{\partial x}\right)+{\left(\frac{1}{1+\kappa y}\right)}^{2}\left(\frac{\partial^{2}v}{\partial x^{2}}-\kappa^{2}v-2\kappa \frac{\partial u}{\partial x}\right)+\frac{1}{1+\kappa y}\kappa \frac{\partial v}{\partial y}+\frac{\partial^{2}v}{\partial y^{2}}\right]\\ \; & +\eta^{o}\left[{\left(\frac{1}{1+\kappa y}\right)}^{3}\frac{\partial \kappa }{\partial x}\left(v-y\frac{\partial u}{\partial x}\right)+{\left(\frac{1}{1+\kappa y}\right)}^{2}\left(\frac{\partial^{2}u}{\partial x^{2}}-\kappa^{2}u+2\kappa \frac{\partial v}{\partial x}\right)+\frac{1}{1+\kappa y}\kappa \frac{\partial u}{\partial y}+\frac{\partial^{2}u}{\partial y^{2}}\right], \end{array}$$

(8) $$\begin{array}{c} \frac{\partial T}{\partial t}+\frac{1}{1+\kappa y}u\frac{\partial T}{\partial x}+v\frac{\partial T}{\partial y}=k_{c}\left[{\left(\frac{1}{1+\kappa y}\right)}^{2}\frac{\partial^{2}T}{\partial x^{2}}-{\left(\frac{1}{1+\kappa y}\right)}^{3}y\frac{\partial \kappa }{\partial x}\frac{\partial T}{\partial x}+\frac{1}{1+\kappa y}\kappa \frac{\partial T}{\partial y}+\frac{\partial^{2}T}{\partial y^{2}}\right], \end{array}$$

where ρ is the liquid density, *p* is the pressure, *T* is the temperature, *g* is the gravitational acceleration, and $k_{c}$ is the thermal diffusivity, which is assumed to be constant.

At the substrate y=0, the boundary conditions for fluid no-slip and no-penetration and the boundary condition for temperature are

(9) u=v=0,

(10) $$T=T_{g}+bx,$$

where $T_{g}$ is the ambient temperature, $b=\Delta T/\hat{\lambda }$ is the linear rate of change of temperature, $\Delta T=T_{H}-T_{C}$, where $T_{H}$ and $T_{C}$ denote the temperatures at the hotter part and colder part of the substrate, respectively.

At the free surface y=h(x,t), dynamic and kinematic boundary conditions are

(11) $$\begin{array}{cc} \; & \frac{1}{\sqrt{1+{\left(\frac{1}{1+\kappa h}h_{x}\right)}^{2}}}[\eta^{e}\left\{{\left(1+\kappa h\right)}^{2}-h_{x}^{2}\right\}\left\{\frac{1}{1+\kappa h}\left(\frac{\partial v}{\partial x}-\kappa u\right)+\frac{\partial u}{\partial y}\right\}+2\eta^{e}\left\{\left(1+\kappa h\right)\frac{\partial v}{\partial y}\right.\\ \; & \left.-\left(\frac{\partial u}{\partial x}+\kappa v\right)\right\}h_{x}+\eta^{o}\left\{{\left(1+\kappa h\right)}^{2}-h_{x}^{2}\right\}\left\{\frac{1}{1+\kappa h}\left(\frac{\partial u}{\partial x}+\kappa v\right)-\frac{\partial v}{\partial y}\right\}+2\eta^{o}\left\{\left(1+\kappa h\right)\frac{\partial u}{\partial y}\right.\\ \; & \left.+\left(\frac{\partial v}{\partial x}-\kappa u\right)\right\}h_{x}]=\left(1+\kappa h\right)\left(\frac{\partial \sigma }{\partial x}+h_{x}\frac{\partial \sigma }{\partial y}\right), \end{array}$$

(12) $$\begin{array}{cc} \; & p_{a}-p+\frac{1}{1+{\left(\frac{1}{1+\kappa h}h_{x}\right)}^{2}}[2\eta^{e}\left\{{\left(\frac{1}{1+\kappa h}\right)}^{3}\left(\frac{\partial u}{\partial x}+\kappa v\right)h_{x}^{2}+\frac{\partial v}{\partial y}-{\left(\frac{1}{1+\kappa h}\right)}^{2}\left(\frac{\partial v}{\partial x}-\kappa u\right)\right.\\ \; & \left.\times h_{x}-\frac{1}{1+\kappa h}\frac{\partial u}{\partial y}h_{x}\right\}+\eta^{o}\left\{\left(1-{\left(\frac{1}{1+\kappa h}h_{x}\right)}^{2}\right)\left(\frac{\partial u}{\partial y}+\frac{1}{1+\kappa h}\left(\frac{\partial v}{\partial x}-\kappa u\right)\right)-\frac{2}{1+\kappa h}\right.\\ \; & \left.\times \left(\frac{1}{1+\kappa h}\left(\frac{\partial u}{\partial x}+\kappa v\right)-\frac{\partial v}{\partial y}\right)h_{x}\right\}]=\sigma \frac{\left(1+\kappa h\right)h_{x\;x}-h\frac{\partial \kappa }{\partial x}h_{x}-\kappa \left[{\left(1+\kappa h\right)}^{2}+2h_{x}^{2}\right]}{{\left[{\left(1+\kappa h\right)}^{2}+h_{x}^{2}\right]}^{3/2}}, \end{array}$$

(13) $$\frac{\partial h}{\partial t}+\frac{1}{1+\kappa h}u\frac{\partial h}{\partial x}-v=0,$$

where σ is the surface tension, and is assumed to vary linearly over a small temperature range

(14) $$\sigma =\sigma_{{}_{0}}-\gamma \left(T-T_{{}_{g}}\right),$$

$\sigma_{{}_{0}}$ is the surface tension at the reference temperature $T_{{}_{g}}$ and $\gamma ={-\partial \sigma /\partial T}_{T=T_{g}}$.

The balance between heat supply to and heat loss at the free surface y=h(x,t) is given by Newton’s law of cooling:

(15) $$\begin{array}{c} -\frac{\lambda }{\sqrt{1+{\left(\frac{1}{1+\kappa h}h_{x}\right)}^{2}}}\left[-\frac{1}{{\left(1+\kappa h\right)}^{2}}h_{x}\frac{\partial T}{\partial x}+\frac{\partial T}{\partial y}\right]=k_{g}\left(T-T_{g}\right), \end{array}$$

where λ is the thermal conductivity, and $k_{g}$ is the heat transfer coefficient between the fluid and the air.

In order to investigate the effect of substrate undulation on the film flow, we use the thin film flow over a flat bottom as referenced. So the Nusselt velocity $u_{0}=\rho gh_{0}{}^{2}\sin\theta /3\eta^{e}$, where $h_{0}$ is the constant film thickness, is also the length scale in the transverse direction, and $\hat{\lambda }$ is the characteristic longitudinal length scale, which is very long compared to the film thickness.

The following dimensionless quantities are introduced to dimensionlessize the equation (indicated by the asterisk):

(16) $$\begin{array}{cc} \; & x^{*}=\frac{2\pi x}{\hat{\lambda }},\;y^{*}=\frac{y}{h_{0}},\;h^{*}=\frac{h}{h_{0}},\;u^{*}=\frac{u}{u_{0}},\;v^{*}=\frac{\hat{\lambda }v}{2\pi h_{0}u_{0}},\\ \; & t^{*}=\frac{2\pi u_{0}t}{\hat{\lambda }},\;p^{*}=\frac{p}{\rho {u_{0}}^{2}},\;\kappa^{*}=\frac{{\hat{\lambda }}^{2}\kappa }{4\pi^{2}\hat{a}},\;T^{*}=\frac{2\pi \left(T-T_{g}\right)}{\Delta T},\\ \; & {\hat{x}}^{*}\left(x^{*}\right)=\frac{2\pi \hat{x}\left(x\right)}{\hat{\lambda }},\;{\hat{b}}^{*}\left({\hat{x}}^{*}\right)=\frac{\hat{b}}{\hat{a}}\left(\frac{\hat{\lambda }{\hat{x}}^{*}}{2\pi }\right),\;\theta^{*}=\arctan\left(\zeta \frac{\partial {\hat{b}}^{*}}{\partial {\hat{x}}^{*}}\right), \end{array}$$

where $\zeta =2\pi \hat{a}/\hat{\lambda }$ is the bottom steepness and $\alpha =2\pi \hat{h}/\hat{\lambda }$ is the aspect ratio.

Using the Equation (16) in the governing equations and boundary conditions, we arrive after dropping the asterisk as

(17) $$\frac{\partial u}{\partial x}+\frac{\partial v}{\partial y}+\alpha \zeta \kappa \left(v+y\frac{\partial v}{\partial y}\right)=0,$$

(18) $$\begin{array}{c} \alpha Re\left(\frac{\partial u}{\partial t}+u\frac{\partial u}{\partial x}+v\frac{\partial u}{\partial y}\right)=-\alpha Re\frac{\partial p}{\partial x}+3\frac{\sin(\beta -\theta )}{\sin\beta }+\frac{\partial^{2}u}{\partial y^{2}}+\alpha \kappa \zeta \frac{\partial u}{\partial y}-\alpha \mu \frac{\partial^{2}v}{\partial y^{2}}+O\left(\alpha^{2}\right), \end{array}$$

(19) $$\begin{array}{c} -\alpha Re\zeta \kappa u^{2}=-Re\frac{\partial p}{\partial y}-3\frac{\cos\left(\beta -\theta \right)}{\sin\beta }+\alpha \frac{\partial^{2}v}{\partial y^{2}}+\mu \left(\alpha \zeta \kappa \frac{\partial u}{\partial y}+\frac{\partial^{2}u}{\partial y^{2}}\right)+O\left(\alpha^{2}\right), \end{array}$$

(20) $$\alpha RePr\left(\frac{\partial T}{\partial t}+u\frac{\partial T}{\partial x}+v\frac{\partial T}{\partial y}\right)=\frac{\partial^{2}T}{\partial y^{2}}+\alpha \zeta \kappa \frac{\partial T}{\partial y}+O\left(\alpha^{2}\right).$$

At the substrate y=0, we have

(21) u=0,v=0,T=x.

At the free surface y=h(x,t), we have

(22) $$\begin{array}{c} \frac{\partial u}{\partial y}+\alpha \zeta \kappa \left(2h\frac{\partial u}{\partial y}-u\right)+\alpha \mu \left\{\left(\frac{\partial u}{\partial x}-\frac{\partial v}{\partial y}\right)+2\frac{\partial u}{\partial y}h_{x}\right\}=-M\;n\left(\frac{\partial T}{\partial x}+h_{x}\frac{\partial T}{\partial y}\right)+O\left(\alpha^{2}\right), \end{array}$$

(23) $$\begin{array}{c} {\bar{p}}_{a}-p+\frac{2\alpha }{Re}\left(\frac{\partial v}{\partial y}-\frac{\partial u}{\partial y}h_{x}\right)+\frac{\mu }{Re}\frac{\partial u}{\partial y}=\alpha^{2}W\;e\left(1-C\;aT\right)\left(h_{x\;x}-\xi \kappa +2\zeta^{2}\kappa^{2}h\right)+O\left(\alpha^{2}\right), \end{array}$$

(24) $$\frac{\partial h}{\partial t}+u\frac{\partial h}{\partial x}-v-\alpha \zeta \kappa hu\frac{\partial h}{\partial x}+O\left(\alpha^{2}\right)=0,$$

(25) $$\frac{\partial T}{\partial y}+BiT+O\left(\alpha^{2}\right)=0,$$

where ${\bar{p}}_{a}=\frac{p_{a}}{\rho {u_{0}}^{2}}$, $\mu =\frac{\eta^{o}}{\eta^{e}}$ is the odd viscosity coefficient, $Re=\frac{\rho u_{\circ }h_{\circ }}{\eta^{e}}$ is the Reynolds number, $W\;e=\frac{\sigma_{0}}{\eta^{e}u_{0}}$ is the Weber number, $M\;n=\frac{\alpha \gamma \Delta T}{\eta^{e}u_{0}}$ is the Marangoni number, $Pr=\frac{\eta^{e}}{\rho k_{c}}$ is the Prandtl number, $C\;a=\frac{\gamma \Delta T}{\sigma_{0}}$ is the Capillary number, and $Bi=\frac{k_{g}h_{0}}{\lambda }$ is the Biot number, $\xi =\frac{\zeta }{\alpha }\equiv \frac{\hat{a}}{\hat{h}}$.

## 3. Approximate Solution of the Equations

The physical quantities *u*, *v*, *p* and *T* are expanded as power series of the small parameter α:

(26) $$\begin{array}{c} u=u^{0}+\alpha u^{1}+\cdots ,\\ v=v^{0}+\alpha v^{1}+\cdots ,\\ p=p^{0}+\alpha p^{1}+\cdots ,\\ T=T^{0}+\alpha T^{1}+\cdots . \end{array}$$

Then we substitute the asymptotic Equation (26) into the dimensionless Equations (17)–(25) to obtain the zero-order governing equations and boundary conditions

(27) $$\frac{\partial u^{0}}{\partial x}+\frac{\partial v^{0}}{\partial y}=0,$$

(28) $$\frac{3\sin\left(\beta -\theta \right)}{\sin\beta }+\frac{\partial^{2}u^{0}}{\partial y^{2}}=0,$$

(29) $$Re\frac{\partial p}{\partial y}=-\frac{3\cos\left(\beta -\theta \right)}{\sin\beta }+\mu \frac{\partial^{2}u^{0}}{\partial y^{2}},$$

(30) $$\frac{\partial^{2}T^{0}}{\partial y^{2}}=0.$$

At the substrate y=0,

(31) $$u^{0}=v^{0}=0,\;T^{0}=x.$$

At the free surface y=h(x,t),

(32) $$\begin{array}{c} \frac{\partial u^{0}}{\partial y}=-M\;n\left(\frac{\partial T^{0}}{\partial x}+h_{x}\frac{\partial T^{0}}{\partial y}\right), \end{array}$$

(33) $$\begin{array}{c} {\bar{p}}_{a}-p^{0}+\frac{\mu }{Re}\frac{\partial u^{0}}{\partial y}=\alpha^{2}W\;e\left(h_{x\;x}-\xi \kappa +2\zeta^{2}\kappa^{2}h\right), \end{array}$$

(34) $$\frac{\partial h}{\partial t}+u^{0}\frac{\partial h}{\partial x}-v^{0}=0,$$

(35) $$\frac{\partial T^{0}}{\partial y}=0.$$

The solutions at zeroth order can be found as

(36) $$u^{0}=\frac{3\sin\left(\beta -\theta \right)}{\sin\beta }\left(hy-\frac{1}{2}y^{2}\right)-M\;ny,$$

(37) $$v^{0}=-\frac{3\sin\left(\beta -\theta \right)}{2\sin\beta }h_{x}y^{2},$$

(38) $$\begin{array}{c} p^{\circ }=\frac{1}{Re}\left(\frac{3\cos\left(\beta -\theta \right)}{\sin\beta }+\mu \frac{3\sin\left(\beta -\theta \right)}{\sin\beta }\right)\left(h-y\right)+{\bar{p}}_{a}-\frac{\mu }{R}e-\alpha^{2}W\;e\left(h_{x\;x}-\xi \kappa +2\zeta^{2}\kappa^{2}h\right). \end{array}$$

The first order governing equations and boundary conditions are obtained.

(39) $$\frac{\partial u^{1}}{\partial x}+\frac{\partial v^{1}}{\partial y}+\zeta \kappa v^{0}+\zeta \kappa y\frac{\partial v^{0}}{\partial y}=0,$$

(40) $$\begin{array}{c} Re\left(\frac{\partial u^{0}}{\partial t}+u^{0}\frac{\partial u^{0}}{\partial x}+v^{0}\frac{\partial u^{0}}{\partial y}\right)=-Re\frac{\partial p^{0}}{\partial x}+\kappa \zeta \frac{\partial u^{0}}{\partial y}-\mu \frac{\partial^{2}v^{0}}{\partial y^{2}}+\frac{\partial^{2}u^{1}}{\partial y^{2}}, \end{array}$$

(41) $$-Re\zeta \kappa u^{0^{2}}=-Re\frac{\partial p^{1}}{\partial y}+\frac{\partial^{2}v^{0}}{\partial y^{2}}+\mu \left(\zeta \kappa \frac{\partial u^{0}}{\partial y}+\frac{\partial^{2}u^{1}}{\partial y^{2}}\right),$$

(42) $$RePr\left(\frac{\partial T^{0}}{\partial t}+u^{0}\frac{\partial T^{0}}{\partial x}+v^{0}\frac{\partial T^{0}}{\partial y}\right)=\frac{\partial^{2}T^{1}}{\partial y^{2}}+\zeta \kappa \frac{\partial T^{0}}{\partial y}.$$

At the substrate y=0,

(43) $$u^{1}=v^{1}=0,\;T^{1}=0.$$

At the free surface y=h(x,t),

(44) $$\begin{array}{c} \frac{\partial u^{1}}{\partial y}+\zeta \kappa \left(2h\frac{\partial u^{0}}{\partial y}-u^{0}\right)+\mu \left\{\left(\frac{\partial u^{0}}{\partial x}-\frac{\partial v^{0}}{\partial y}\right)+2\frac{\partial u^{0}}{\partial y}h_{x}\right\}=-M\;n\left(\frac{\partial T^{1}}{\partial x}+h_{x}\frac{\partial T^{1}}{\partial y}\right), \end{array}$$

(45) $$-p^{1}+\frac{2}{Re}\left(\frac{\partial v^{0}}{\partial y}-\frac{\partial u^{0}}{\partial y}h_{x}\right)+\frac{\mu }{Re}\frac{\partial u^{1}}{\partial y}=0,$$

(46) $$u^{1}\frac{\partial h}{\partial t}-v^{1}-\zeta \kappa hu^{0}\frac{\partial h}{\partial x}=0,$$

(47) $$\frac{\partial T^{1}}{\partial y}=0.$$

By solving the first order equation, we obtain the expression of $T^{1}$ and $u^{1}$ as

(48) $$\begin{array}{cc} T^{1}=RePr\{ & \frac{\sin\left(\beta -\theta \right)}{\sin\beta }\left(-\frac{1}{8}y^{4}+\frac{1}{2}hy^{3}-h^{3}y\right)-M\;n\left(\frac{1}{6}y^{3}-\frac{1}{2}h^{2}y\right)\}, \end{array}$$

(49) $$\begin{array}{cc} u^{1}= & \frac{1}{2}Re\frac{\sin\left(\beta -\theta \right)}{\sin\beta }\left(y^{3}-3h^{2}y\right)h_{t}+M\;nRePr\left(\frac{5}{2}\frac{\sin\left(\beta -\theta \right)}{\sin\beta }h^{3}-M\;nh^{2}\right)h_{x}y+Re\\ \; & \times \frac{\sin\left(\beta -\theta \right)}{\sin\beta }\left\{\frac{3}{8}\frac{\sin\left(\beta -\theta \right)}{\sin\beta }\left(y^{3}-4h^{3}\right)hy-\frac{1}{8}M\;n\left(y^{3}-4h^{3}\right)y\right\}h_{x}+Re\left(\frac{1}{2}y^{2}\right.\\ \; & \left.-hy\right)\left\{\frac{3\cos\left(\beta -\theta \right)}{Re\sin\beta }h_{x}-\alpha^{2}W\;e\left(h_{x\;x\;x}-\xi \frac{\partial \kappa }{\partial x}+2\zeta^{2}\kappa^{2}h_{x}+4\zeta^{2}\kappa h\frac{\partial \kappa }{\partial x}\right)\right\}+\zeta \kappa \\ \; & \times \left\{\frac{\sin\left(\beta -\theta \right)}{\sin\beta }\left(\frac{1}{2}y^{3}-\frac{3}{2}hy^{2}+3h^{2}y\right)+\frac{1}{2}M\;ny^{2}\right)-2\mu \left(\frac{3\sin\left(\beta -\theta \right)}{\sin\beta }h-M\;n\right)h_{x}y. \end{array}$$

From the continuity equation, we have

(50) $$h_{t}=-\left(\frac{3\sin\left(\beta -\theta \right)}{\sin\beta }h^{2}-M\;nh\right)h_{x}.$$

Putting this in (49), we obtain

(51) $$\begin{array}{cc} u^{1}= & M\;nRePr\left(\frac{5}{2}\frac{\sin\left(\beta -\theta \right)}{\sin\beta }h^{3}-M\;nh^{2}\right)h_{x}y+Re\frac{\sin\left(\beta -\theta \right)}{\sin\beta }\{\frac{\sin\left(\beta -\theta \right)}{\sin\beta }\left(\frac{3}{8}hy^{4}-\frac{3}{2}h^{2}y^{3}\right.\\ \; & \left.+3h^{4}y\right)+M\;n\left(-\frac{1}{8}y^{4}+\frac{1}{2}hy^{3}-h^{3}y\right)\}h_{x}+Re\left(\frac{1}{2}y^{2}-hy\right)\{\frac{3\cos(\beta -\theta )}{Re\sin\beta }h_{x}\\ \; & -\alpha^{2}W\;e\left(h_{x\;x\;x}-\xi \frac{\partial \kappa }{\partial x}+2\zeta^{2}\kappa^{2}h_{x}+4\zeta^{2}\kappa h\frac{\partial \kappa }{\partial x}\right)\}+\zeta \kappa \{\frac{\sin\left(\beta -\theta \right)}{\sin\beta }\left(\frac{1}{2}y^{3}-\frac{3}{2}hy^{2}+3h^{2}y\right)\\ \; & +\frac{1}{2}M\;ny^{2}\}-2\mu \left(\frac{3\sin\left(\beta -\theta \right)}{\sin\beta }h-M\;n\right)h_{x}y. \end{array}$$

The local flow rate q(x,t) is defined in

(52) $$q\left(x,t\right)=\int_{0}^{h}u\left(x,y,t\right)dy,$$

where $u\left(x,y,t\right)=u^{0}\left(x,y,t\right)+\alpha u^{1}\left(x,y,t\right)+O\left(\alpha^{2}\right)$. Solving for the integral on the right-hand side of Equation (50), we obtain

(53) $$\begin{array}{cc} q(x,t)= & \frac{\sin\left(\beta -\theta \right)}{\sin\beta }h^{3}-\frac{1}{2}M\;nh^{2}+\alpha [M\;nRe\left(\frac{5}{4}\frac{\sin\left(\beta -\theta \right)}{\sin\beta }h^{5}-\frac{1}{2}M\;nh^{4}\right)h_{x}+Re\frac{\sin\left(\beta -\theta \right)}{\sin\beta }\\ \; & \times \left(\frac{6}{5}\frac{\sin\left(\beta -\theta \right)}{\sin\beta }h^{6}-\frac{2}{5}M\;nh^{5}\right)h_{x}-\frac{Re}{3}\{\frac{3\cos\left(\beta -\theta \right)}{Re\sin\beta }h_{x}-\alpha^{2}W\;e(h_{x\;x\;x}-\xi \frac{\partial \kappa }{\partial x}\\ \; & +2\zeta^{2}\kappa^{2}h_{x}+4\zeta^{2}\kappa h\frac{\partial \kappa }{\partial x})\}h^{3}+\zeta \kappa \left(\frac{9}{8}\frac{\sin(\beta -\theta )}{\sin\beta }h^{4}+\frac{1}{6}M\;nh^{3}\right)\\ \; & -\mu \left(\frac{3\sin(\beta -\theta )}{\sin\beta }h^{3}-M\;nh^{2}\right)h_{x}]. \end{array}$$

Using alternative form of the kinematic boundary conditions

(54) $$\frac{\partial h}{\partial t}+\left(1-\alpha \zeta \kappa h\right)\frac{\partial q}{\partial x}+O\left(\alpha^{2}\right)=0.$$

We obtain a non-linear evolution equation for the thickness of the film

(55) $$h_{t}+A\left(h\right)h_{x}+\alpha {\left(B\left(h\right)h_{x}+C\left(h\right)h_{x\;x\;x}\right)}_{x}=0,$$

where,

(56) $$\begin{array}{cc} A(h)= & \left(\frac{3\sin\left(\beta -\theta \right)}{\sin\beta }h-M\;n\right)h+\alpha \{\alpha^{2}W\;eRe\left(-\xi +\frac{16}{3}\zeta^{2}\kappa h\right)h^{2}\frac{\partial \kappa }{\partial x}\\ \; & +\frac{3}{2}\zeta \kappa h^{2}\left(\frac{\sin(\beta -\theta )}{\sin\beta }h+M\;n\right)\}, \end{array}$$

(57) $$\begin{array}{cc} B(h)= & M\;nRePr\left(\frac{5}{4}\frac{\sin\left(\beta -\theta \right)}{\sin\beta }h^{5}-\frac{1}{2}M\;nh^{4}\right)+Re\frac{\sin\left(\beta -\theta \right)}{\sin\beta }\left(\frac{6}{5}\frac{\sin\left(\beta -\theta \right)}{\sin\beta }h^{6}-\frac{2}{5}M\;nh^{5}\right)\\ \; & -\left(\frac{\cos\left(\beta -\theta \right)}{\sin\beta }-\frac{2}{3}\alpha^{2}W\;eRe\zeta^{2}\kappa^{2}\right)h^{3}-\mu \left(\frac{3\sin\left(\beta -\theta \right)}{\sin\beta }h^{3}-M\;nh^{2}\right), \end{array}$$

(58) $$C\left(h\right)=\frac{1}{3}\alpha^{2}W\;eReh^{3}.$$

We introduce a new parameter *S*, as defined by earlier researchers [37]:

(59) $$S=\alpha^{2}W\;e.$$

From now on, for the sake of simplicity of the formula, we shall use the following abbreviation:

(60) $$s=\frac{\sin(\beta -\theta )}{\sin\beta },\;c=\frac{\cos(\beta -\theta )}{\sin\beta }.$$

## 4. Linear Stability Analysis

To investigate the stability of the thin film flow, a small perturbation at the free interface is assumed. The film thickness *h*, which can be written as

(61) h=1+η(x,t),

where η≪1 denotes the dimensionless distance of free surface of the liquid film from free surface of the smooth laminar flow.

To eliminate the α in Equation (55), we set the conversion

(62) $$x=\alpha \tilde{x},\;t=\alpha \tilde{t}.\;$$

Substituting Equations (61) and (62) into Equation (55), retaining up to $O(\eta^{3})$, after dropping the cap sign, we obtain

(63) $$\begin{array}{cc} & \eta_{t}+A_{1}\eta_{x}+B_{1}\eta_{x\;x}+C_{1}\eta_{x\;x\;x\;x}+A_{1}^{\prime }\eta \eta_{x}+B_{1}^{\prime }\left(\eta \eta_{x\;x}+\eta_{x}^{2}\right)+C_{1}^{\prime }\left(\eta \eta_{x\;x\;x\;x}+\eta_{x}\eta_{x\;x\;x}\right)\\ \; & +\frac{1}{2}A_{1}^{\prime \prime }\eta^{2}\eta_{x}+B_{1}^{\prime \prime }\left(\frac{1}{2}\eta^{2}\eta_{x\;x}+\eta \eta_{x}^{2}\right)+C_{1}^{\prime \prime }\left(\frac{1}{2}\eta^{2}\eta_{x\;x\;x\;x}+\eta \eta_{x}\eta_{x\;x\;x}\right)+O\left(\eta^{4}\right)=0, \end{array}$$

where $A_{1},B_{1},C_{1}$ and their corresponding derivatives (denoted by primes) are the values corresponding to h=1. The linear response of the film is studied by assuming that the perturbation is in the form of a sinusoidal perturbation, that is

(64) η(x,t)=Γexp[i(kx−ωt)]+c.c.,

where Γ is the amplitude of the disturbance, *k* is the wave number, c.c. is the complex conjugate and $\omega =\omega_{r}+i\omega_{i}$ is the complex frequency, $\omega_{r}$ and $\omega_{i}$ are the linear growth rates of oscillation frequency and amplitude, respectively. Substituting Equation (64) into Equation (63) and considering the linear part, the dispersion relation can be obtained as

(65) $$\mathrm{Disp}\left(\omega ,k\right)=-i\omega +iA_{1}k-B_{1}k^{2}+C_{1}k^{4}=0.$$

In Equation (65), the real and the imaginary parts of ω are expressed as

(66) $$\begin{array}{cc} \; & \omega_{r}=A_{1}k,\\ \; & \omega_{i}=B_{1}k^{2}-C_{1}k^{4}. \end{array}$$

The flow will be linearly unstable if the linear growth rate of amplitude is $\omega_{i}>0$, conversely, the flow is linearly stable, and the flow will be neutrally stable if $\omega_{i}=0$. At this point, the critical Reynolds number is

(67) $$Re_{c}=\frac{c-2B\;o\zeta^{2}\kappa^{2}+\mu \left(3s-M\;n\right)}{\frac{1}{4}M\;nPr\left(5s-2M\;n\right)+\frac{2}{5}s\left(3s-M\;n\right)}\;,$$

where $B\;o=4\pi^{2}\sigma_{0}/\rho g{\hat{\lambda }}^{2}\sin\beta$ is the inverse Bond number and 3Bo=SRe.

As μ→0, we obtained the same critical Reynolds number as that derived by Mukhopadhyay and Mukhopadhyay [35].

## 5. Weakly Non-Linear Analysis

We use the method of multiple scale and expend the interfacial perturbation η in the following form [38,39,40]

(68) $$\eta \left(x,x_{1},\ldots ,t,t_{1},t_{2},\ldots \right)=\varepsilon \eta_{11}+\varepsilon^{2}\eta_{12}+\varepsilon^{3}\eta_{13}+\ldots ,$$

where

(69) $$\begin{array}{c} x_{1}=\varepsilon x,t_{1}=\varepsilon t,t_{2}=\varepsilon^{2}t,\ldots , \end{array}$$

here *x* and *t* represent the rapidly varying scales, while $x_{1},t_{1}$, and so on represent the slowly varying scales. Assuming these variables are independent of each other, then the derivatives of time and space become as shown in

(70) $$\partial_{t}\to \partial_{t}+\varepsilon \partial_{t_{1}}+\varepsilon^{2}\partial_{t_{2}},$$

(71) $$\partial_{x}\to \partial_{x}+\varepsilon \partial_{x_{1}}.$$

Substituting the Equations (68)–(71) into the Equation (63), we obtain

(72) $$\begin{array}{c} \left(L_{0}+\varepsilon L_{1}+\varepsilon^{2}L_{2}+\ldots \right)\left(\varepsilon \eta_{11}+\varepsilon^{2}\eta_{12}+\varepsilon^{3}\eta_{13}+\ldots \right)=-\varepsilon^{2}N_{2}-\varepsilon^{3}N_{3}-\ldots , \end{array}$$

where the operators $L_{0},L_{1},L_{2}$, and the non-linear terms $N_{2},N_{3}$ in Equation (72) are shown below.

(73) $$\begin{array}{cc} L_{0}= & \frac{\partial }{\partial t}+A_{1}\frac{\partial }{\partial x}+B_{1}\frac{\partial^{2}}{\partial x^{2}}+C_{1}\frac{\partial^{4}}{\partial x^{4}},\\ L_{1}= & \frac{\partial }{\partial t_{1}}+A_{1}\frac{\partial }{\partial x_{1}}+2B_{1}\frac{\partial^{2}}{\partial x\partial x_{1}}+4C_{1}\frac{\partial^{4}}{\partial x^{3}\partial x_{1}},\\ L_{2}= & \frac{\partial }{\partial t_{2}}+B_{1}\frac{\partial^{2}}{\partial x_{1}^{2}}+6C_{1}\frac{\partial^{4}}{\partial x^{2}\partial x_{1}^{2}},\\ N_{2}= & A^{\prime }\eta_{11}\frac{\partial \eta_{11}}{\partial x}+B_{1}^{\prime }\left[\eta_{11}\frac{\partial^{2}\eta_{11}}{\partial x^{2}}+{\left(\frac{\partial \eta_{11}}{\partial x}\right)}^{2}\right]+C_{1}^{\prime }\left[\eta_{11}\frac{\partial^{4}\eta_{11}}{\partial x^{4}}+\frac{\partial \eta_{11}}{\partial x}\frac{\partial^{3}\eta_{11}}{\partial x^{3}}\right],\\ N_{3}= & A_{1}^{\prime }\left[\eta_{11}\left(\frac{\partial \eta_{12}}{\partial x}+\frac{\partial \eta_{11}}{\partial x_{1}}\right)+\eta_{12}\frac{\partial \eta_{11}}{\partial x}\right]+B_{1}^{\prime }[\eta_{11}\left(\frac{\partial^{2}\eta_{12}}{\partial x^{2}}+2\frac{\partial^{2}\eta_{11}}{\partial x\partial x_{1}}\right)+\eta_{12}\frac{\partial^{2}\eta_{11}}{\partial x^{2}}\\ \; & +2\frac{\partial \eta_{11}}{\partial x}\left(\frac{\partial \eta_{12}}{\partial x}+\frac{\partial \eta_{11}}{\partial x_{1}}\right)]+C_{1}^{\prime }[\eta_{11}\left(\frac{\partial^{4}\eta_{12}}{\partial x^{4}}+4\frac{\partial^{4}\eta_{11}}{\partial x^{3}\partial x_{1}}\right)+\eta_{12}\frac{\partial^{4}\eta_{11}}{\partial x^{4}}+\frac{\partial \eta_{11}}{\partial x}\\ \; & \times \left(\frac{\partial^{3}\eta_{12}}{\partial x^{3}}+3\frac{\partial^{3}\eta_{11}}{\partial x^{2}\partial x_{1}}\right)+\frac{\partial^{3}\eta_{11}}{\partial x^{3}}\left(\frac{\partial \eta_{11}}{\partial x_{1}}+\frac{\partial \eta_{12}}{\partial x}\right)]+\frac{1}{2}A_{1}^{\prime \prime }\eta_{11}^{2}\frac{\partial \eta_{11}}{\partial x}\\ \; & +B_{1}^{\prime \prime }\left[\frac{1}{2}\eta_{11}^{2}\frac{\partial^{2}\eta_{11}}{\partial x^{2}}+\eta_{11}{\left(\frac{\partial \eta_{11}}{\partial x}\right)}^{2}\right]. \end{array}$$

For the first-order equation of ε, we have

(74) $$L_{0}\eta_{11}=0,$$

the solution of this equation has the following form:

(75) $$\eta_{{}_{11}}=\Gamma \left(x_{{}_{1}},t_{{}_{1}},t_{{}_{2}}\right)\left[\exp i\Theta \right]+c.c.,$$

where $\Gamma (x_{{}_{1}},t_{{}_{1}},t_{{}_{2}})$ is the complex amplitude. $\Theta =kx-\omega_{r}t$, $\omega =\omega_{r}+i\omega_{i}$ is the complex frequency, because near the neutral curve $\omega_{i}∼O\left(\zeta^{2}\right)$. Thus, the function $\exp(\omega_{i}t)$ is slow and may be absorbed in $\Gamma (x_{1},t_{1},t_{2})$.

For the second-order equation of ε, we have

(76) $$L_{0}\eta_{12}=-L_{1}\eta_{11}-N_{2}.$$

Substituting the expression (75) into the Equation (76), we obtain

(77) $$\begin{array}{cc} L_{0}\eta_{12}= & -i\left[\frac{\partial \mathrm{Disp}(\omega_{r},k)}{\partial \omega_{r}}\frac{\partial \Gamma }{\partial t_{1}}-\frac{\partial \mathrm{Disp}(\omega_{r},k)}{\partial k}\frac{\partial \Gamma }{\partial x_{1}}\right]e^{i\Theta }-Q_{1}\Gamma^{2}e^{2i\Theta }+c.c., \end{array}$$

where $\mathrm{Disp}(\omega_{r},k)$ is given by Equation (65) and

(78) $$Q_{1}=iA_{1}^{\prime }k-2B_{1}^{\prime }k^{2}+2C_{1}^{\prime }k^{4}.$$

The solution $\eta_{12}$ is obtained from Equation (77) in the following form:

(79) $$\eta_{12}=-\frac{Q_{1}\Gamma^{2}e^{2i\Theta }}{\mathrm{Disp}(2\omega_{r},2k)}+c.c..$$

Introducing the coordinate transformation $\delta =\left(x_{1}-c_{g}t_{1}\right)$, where $c_{g}=\left(-{\mathrm{Disp}}_{k}/{\mathrm{Disp}}_{\omega_{r}}\right)$, and using the solvability condition of the third-order equation, we obtain

(80) $$\frac{\partial \Gamma }{\partial t_{2}}+J_{1}\frac{\partial^{2}\Gamma }{\partial \delta^{2}}-\varepsilon^{-2}\omega_{i}\Gamma +\left(J_{2}+iJ_{3}\right){\left|\Gamma \right|}^{2}\Gamma =0,$$

where

(81) $$\begin{array}{cc} J_{1}= & B_{1}-6C_{1}k^{2},\\ J_{2}= & \frac{1}{2}\left(C_{1}^{\prime \prime }k^{4}-B_{1}^{\prime \prime }k^{2}\right)+\frac{{\left(A_{1}^{\prime }\right)}^{2}k^{2}+2\left(7C_{1}^{\prime }k^{4}-B_{1}^{\prime }k^{2}\right)\left(B_{1}^{\prime }k^{2}-C_{1}^{\prime }k^{4}\right)}{16C_{1}k^{4}-4B_{1}k^{2}},\\ J_{3}= & \frac{1}{2}A_{1}^{\prime \prime }k+\frac{A_{1}^{\prime }k\left(B_{1}^{\prime }k^{2}-7C_{1}^{\prime }k^{4}\right)+2A_{1}^{\prime }k\left(B_{1}^{\prime }k^{2}-C_{1}^{\prime }k^{4}\right)}{16C_{1}k^{4}-4B_{1}k^{2}}. \end{array}$$

The diffusion effect in Equation (80) is neglected to obtain

(82) $$\frac{\partial \Gamma }{\partial t_{2}}-\varepsilon^{-2}\omega_{i}\Gamma +\left(J_{2}+iJ_{3}\right){\left|\Gamma \right|}^{2}\Gamma =0.$$

The solution of the equation can be written as

(83) $$\Gamma =a\exp[-ib\left(t_{2}\right)t_{2}].$$

Substituting Equation (83) into Equation (82), we obtain

(84) $$\frac{\partial a}{\partial t_{2}}=\left(\varepsilon^{-2}\omega_{i}-J_{2}a^{2}\right)a,$$

(85) $$\frac{\partial [b\left(t_{2}\right)t_{2}]}{\partial t_{2}}=J_{3}a^{2}.$$

The second term on the right-hand side of Equation (84) is a non-linear term that regulates the exponential change of the linear perturbation, depending on the sign of $\omega_{i}$ and $J_{2}$. When the right side of Equation (84) is 0, the equilibrium amplitude is solved as

(86) $$\varepsilon a=\sqrt{\frac{\omega_{i}}{J_{2}}}.$$

The term $J_{{}_{2}}<0$ can lead to the instability of the system. According to the signs of $\omega_{i}$ and $J_{2}$, four non-linear regions are defined, which are: the supercritical stability region $I(\omega_{i}>0,J_{2}>0)$, the subcritical instability region $II(\omega_{i}<0,J_{2}<0)$, the unconditionally stable region $III(\omega_{i}<0,J_{2}>0)$ and the explosive state region $IV(\omega_{i}>0,J_{2}<0)$.

## 6. Numerical Simulations

We numerically solve Equation (55) within a periodic domain to comprehend the evolution of finite amplitude perturbations. To achieve this, we transform the problem into a set of ordinary differential equations (ODE) by discretizing the spatial variables into a series of wave numbers through the Fourier transform. Subsequently, we employ the fast Fourier transform algorithm to compute these wave numbers as the numerical solution to the nonlinear evolution Equation (55) [41].

Fourier transform of the Equation (55) in the *x* domain

(87) $$\begin{array}{cc} \frac{\partial \hat{h}}{\partial t}= & -ik\cdot F\{sF^{-1}{\left[\hat{h}\right]}^{3}-\frac{1}{2}M\;nF^{-1}{\left[\hat{h}\right]}^{2}+\zeta \kappa \left(\frac{3}{8}sF^{-1}{\left[\hat{h}\right]}^{3}\right)-SRe(\frac{1}{3}\xi F^{-1}{\left[\hat{h}\right]}^{3}-\frac{4}{3}\zeta^{2}\kappa \\ \; & \times F^{-1}{\left[\hat{h}\right]}^{4})\frac{\partial \kappa }{\partial x}+M\;nRePr\left(\frac{5}{4}sF^{-1}{\left[\hat{h}\right]}^{5}-\frac{1}{2}M\;nF^{-1}{\left[\hat{h}\right]}^{4}\right)F^{-1}\left[ik\hat{h}\right]+Res\left(\frac{6}{5}s\right.\\ \; & \times \left.F^{-1}{\left[\hat{h}\right]}^{6}-\frac{2}{5}M\;nF^{-1}{\left[\hat{h}\right]}^{5}\right)F^{-1}\left[ik\hat{h}\right]-cF^{-1}{\left[\hat{h}\right]}^{3}F^{-1}\left[ik\hat{h}\right]+\frac{2}{3}SRe\zeta^{2}\kappa^{2}F^{-1}{\left[\hat{h}\right]}^{3}\\ \; & \times F^{-1}\left[ik\hat{h}\right]-\mu \left(3sF^{-1}{\left[\hat{h}\right]}^{3}-M\;nF^{-1}{\left[\hat{h}\right]}^{2}\right)F^{-1}\left[ik\hat{h}\right]+\frac{1}{3}SReF^{-1}{\left[\hat{h}\right]}^{3}F^{-1}\left[{\left(ik\right)}^{3}\hat{h}\right]\}. \end{array}$$

Initially, a finite-amplitude monochromatic disturbance is given as

(88) h(x,0)=1+0.03coskx,

where *k* is the wave number. The computation is performed on a uniform grid with the number of spatial grid points varying N = 200 and Δt=0.1∼0.2.

## 7. Specific Case Study

We choose the substrate profile as

(89) $$\hat{b}\left(\hat{x}\right)=\hat{a}\sin\left(2\hat{x}/\hat{\lambda }\right),$$

where $\hat{\lambda }=3$ is the wavelength and $\hat{a}=0.15$ is the amplitude of the wavy bottom profile [35]. $\hat{x}=10\pi /3$ is a point on the “uphill” and $\hat{x}=5\pi /3$ is a point on the “downhill”. We will understand more easily according to Figure 1. Moreover, the basal steepness ζ is a fixed quantity rather than a perturbation parameter. Since both the basal curvature $\kappa (\hat{x})$ and the local inclination $\theta (\hat{x})$ are functions of $\hat{x}$, the critical Reynolds number is also a function of $\hat{x}$.

## 8. Results and Discussion

### 8.1. Linear Stability Analysis

**Figure 2:**
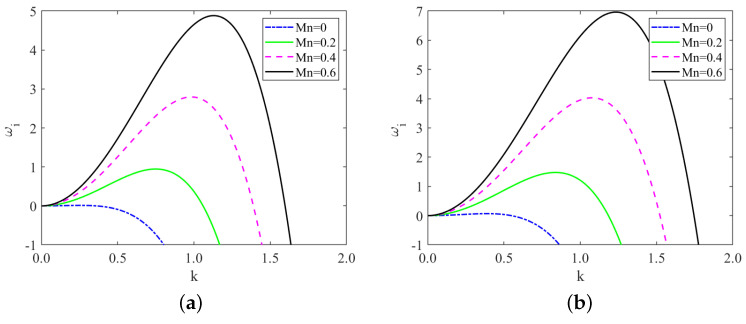
Temporal growth rate curve derived from $\omega_{i}=B_{1}k^{2}-C_{1}k^{4}$, (**a**) x=10π/3; (**b**) x=5π/3, for different values of Marangoni number when Re=2, ζ=0.1π, S=4.5, Pr=7, μ=0.4, β=π/3.

Figure 2 depicts the temporal growth rate curve for various values of the Marangoni number. It is evident that the Marangoni number exerts a destabilizing effect on the system. The destabilizing role of the Marangoni number Mn can be explained from a physical perspective as follows: when the liquid film flows downward along the substrate, the thermocapillary force appearing at the interface acts in the opposite direction of the gravitational acceleration as Mn increases and then enhances the growth of the surface instabilities [42].

**Figure 3:**
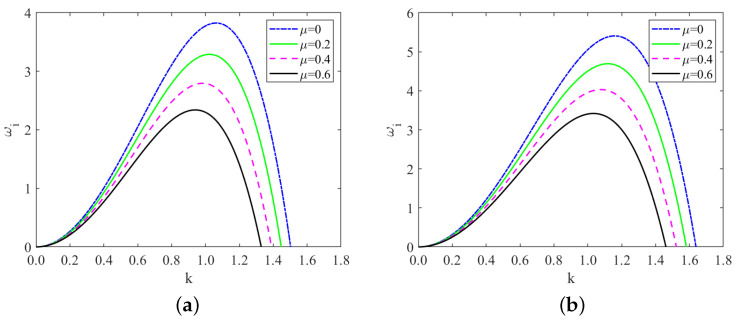
Temporal growth rate curve derived from $\omega_{i}=B_{1}k^{2}-C_{1}k^{4}$, (**a**) x=10π/3; (**b**) x=5π/3, for different values of odd viscosity when Re=2, ζ=0.1π, S=4.5, Pr=7, Mn=0.4, β=π/3.

Figure 3 displays the temporal growth rate curve for different values of odd viscosity. Notably, the odd viscosity μ has a stabilizing effect on the system. This is because the odd viscosity μ comes into play effectively through shear stress on the free surface as the liquid film flows downward along the substrate, causing additional stress on the free surface, which increases the critical Reynolds number giving the stabilizing effect [32]. Equation (67) further supports this observation, showing that the critical Reynolds number $Re_{c}$ rises as the odd viscosity μ increases. As a result, higher values of odd viscosity lead to a more stable fluid flow, reducing the likelihood of surface perturbations and instability. In addition, we compare Figure 2a,b, or Figure 3a,b: interestingly, the fluid exhibits greater stability in the “uphill” part under the same conditions. This distinction is attributed to the difference in the sign of the local inclination angle θ in these regions. The variation in θ plays a significant role in influencing the stability characteristics of the fluid flow along the substrate.

**Figure 4:**
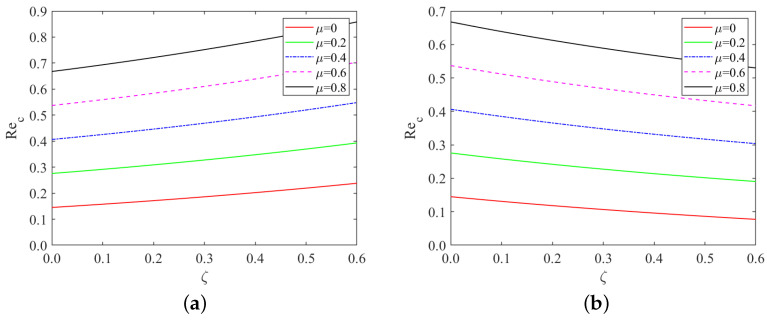
Critical Reynolds number as a function of bottom steepness, (**a**) x=10π/3; (**b**) x=5π/3, for different values of odd viscosity when Re=2, ζ=0.1π, S=4.5, Pr=7, Mn=0.4, β=π/3.

In Figure 4, it is evident that under same conditions, the critical Reynolds number of the “uphill” point increases with an increase in the bottom steepness ζ. In contrast, the critical Reynolds number of the “downhill” point decreases as the bottom steepness ζ increases. From Equation (67), for a positive local inclination angle θ, an increase in bottom steepness ζ inhibits the instability of the liquid film flow. Conversely, with a negative local inclination angle θ, increased bottom steepness ζ promotes the instability of the liquid film flow.

**Figure 5:**
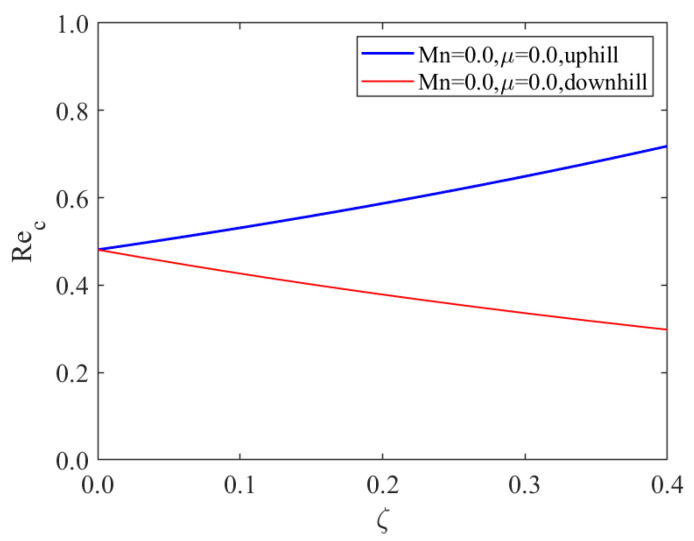
Comparison of $Re_{c}$ as a function of bottom steepness for isothermal bottom at a point on the “downhill” (x=5π/3) and at a point on the “uphill” (x=10π/3) portion when Bo=1, Pr=7, β=π/3.

Moreover, as Mn→0, μ→0 and ζ→0, we find from Figure 5 that the value of the critical Reynolds number is equal to ${Re}_{c}=\left(5/6\right)\cot\beta ∼0.48$, which was originally obtained by Benjamin [7] and Yih [8].

### 8.2. Weakly Non-Linear Stability Analysis

**Figure 6:**
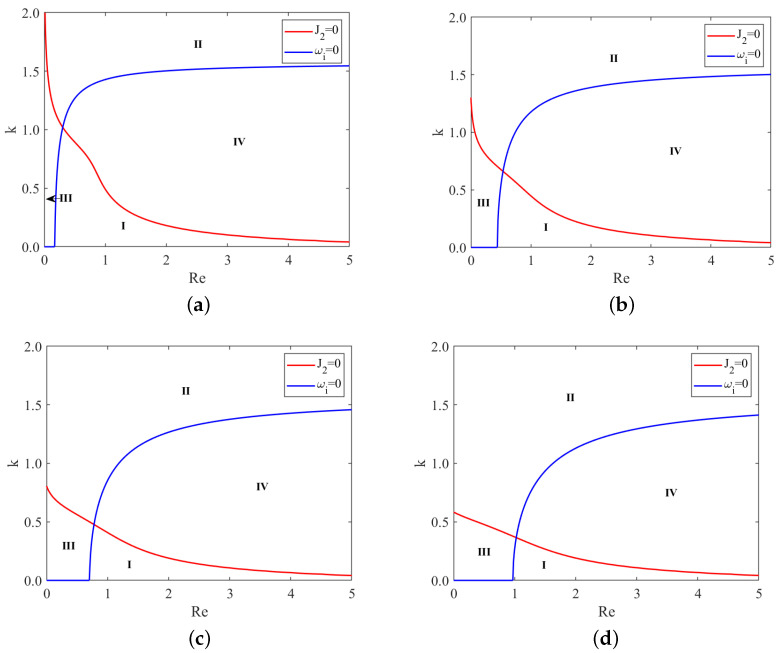
Stability curves, (**a**) μ=0; (**b**) μ=0.4; (**c**) μ=0.8; (**d**) μ=1.2, for different odd viscosity μ when ζ=0.1π, S=4.5, Pr=7, Mn=0.4, β=π/3. I–IV represent the supercritical stability region, the subcritical instability region, unconditional stability region and the explosive region, respectively.

From Figure 6, we observe that, under same conditions, the subcritical instability region II and unconditional stability region III gradually enlarge as the odd viscosity μ increases. In contrast, the supercritical stability region I and the explosive region IV gradually shrink. Moreover, the critical Reynolds number $Re_{c}$ increases with the odd viscosity μ. This implies that the odd viscosity has a stabilizing effect on the flow, as it leads to larger stability regions and higher critical Reynolds numbers.

**Figure 7:**
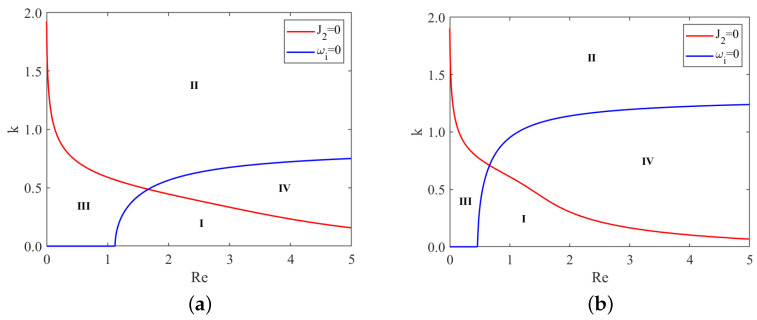

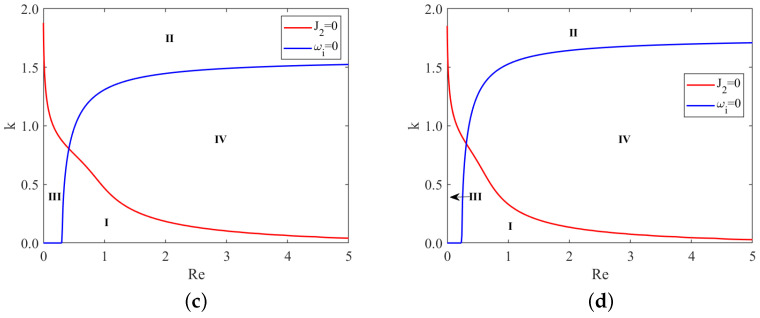
Stability curves, (**a**) Mn=0; (**b**) Mn=0.2; (**c**) Mn=0.4; (**d**) Mn=0.6, for different Marangoni number Mn when ζ=0.1π, S=4.5, Pr=7, μ=0.2, β=π/3. I–IV represent the supercritical stability region, the subcritical instability region, unconditional stability region and the explosive region, respectively.

Similarly, Figure 7 reveals that, under same conditions, the subcritical instability region II and unconditional stability region III gradually shrink as the Marangoni number Mn increases. Conversely, the supercritical stability region I and the explosive region IV gradually enlarge. Additionally, the critical Reynolds number $Re_{c}$ decreases with the increase in the Marangoni number. Hence, the Marangoni effect destabilizes the flow, resulting in reduced stability regions and lower critical Reynolds numbers.

### 8.3. Numerical Simulations

**Figure 8:**
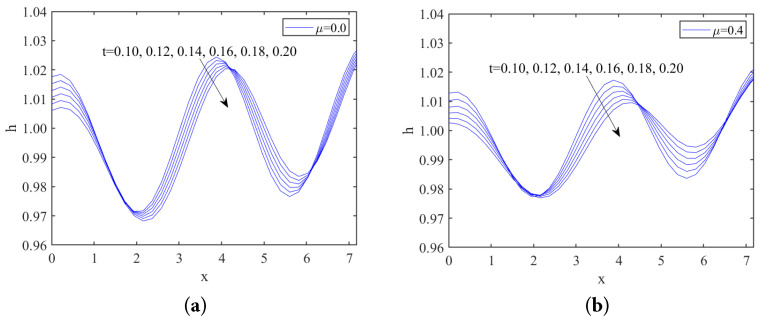
Film thickness at different times, (**a**) μ=0; (**b**) μ=0.4, when Re=2, k=1.75, ζ=0.1π, S=4.5, Pr=7, Mn=0.5, β=π/3.

**Figure 9:**
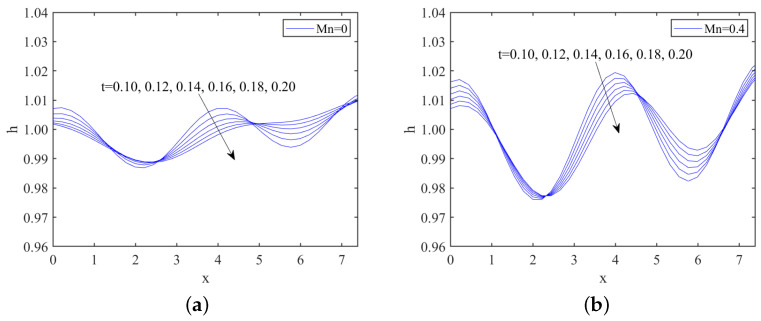
Film thickness at different times, (**a**) Mn=0; (**b**) Mn=0.4, when Re=1, k=1.7, ζ=0.1π, S=4.5, Pr=7, μ=0.2, β=π/3.

Figure 8 and Figure 9 illustrate the short-time evolution of the free surface for different values of odd viscosity μ and the Marangoni number Mn in the subcritical instability region, respectively. As shown in both figures, the disturbance amplitude gradually decreases over time, indicating that the liquid film stabilizes as the disturbances dampen. This behavior is characteristic of the subcritical instability region, where disturbances do not grow unbounded but rather stabilize over time. Furthermore, when comparing Figure 8 and Figure 9, we can observe that the odd viscosity μ has a stabilizing effect. Conversely, the Marangoni number Mn has a destabilizing effect.

**Figure 10:**
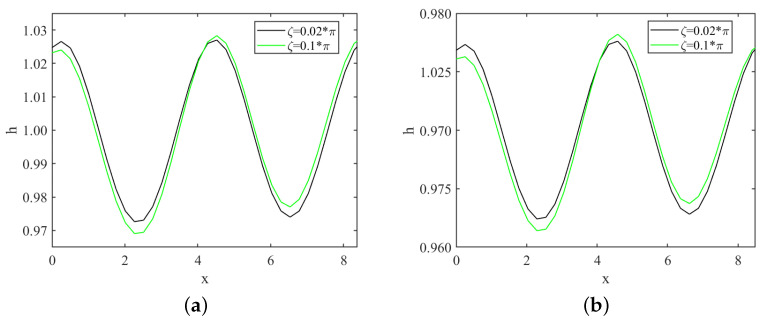
Variations of waves with bottom steepness through numerical simulation, (**a**) when Re=1, k=1.5, S=4.5, Pr=7, μ=0.2, Mn=0.4, β=π/3; (**b**) Re=3, k=1.48, S=4.5, Pr=7, μ=0.2, Mn=0.4, β=π/3.

**Figure 11:**
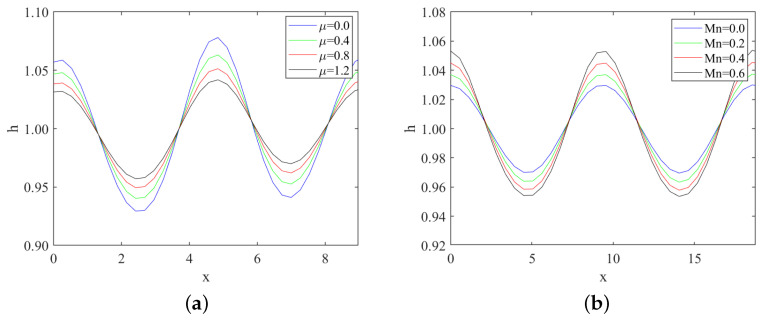
Variations of waves with different odd viscosity and Marangoni number through numerical simulations, (**a**) odd viscosity μ when Re=3, k=1.4, S=4.5, Pr=7, Mn=0.5, β=π/3; (**b**) Marangoni number Mn when Re=3, k=0.67, S=4.5, Pr=7, μ=0.2, β=π/3.

In Figure 10a, we can see that under the same conditions, the amplitude of the free surface disturbance first becomes smaller and then larger as the bottom steepness ζ increases in the subcritical instability region. This observation supports the previous conclusion that the critical Reynolds number of the “uphill” point increases with the bottom steepness ζ, while the “downhill” point decreases with increasing ζ. The varying amplitudes of the free surface disturbance further highlight the contrasting roles of the bottom steepness ζ in influencing the stability of the fluid flow at different regions of the substrate. We can observe wave changes within the explosive region, as depicted in Figure 10b and Figure 11. The obtained results align with those within the subcritical unstable region.

## 9. Conclusions

We have mainly studied the effect of odd viscosity on the instability of falling liquid film over a non-uniformly heated inclined corrugated substrate. To simplify the analysis, we neglect the evaporation effect by assuming a nonvolatile fluid. Additionally, for the sake of convenience, we consider the free surface to be adiabatic. The mechanism of thermocapillary helps move the fluid from a warmer region to a colder neighborhood. This study is conducted on general periodic bottom contours and is analyzed and discussed in the specific case of sinusoidal bottoms.

In our investigation, we carefully considered the impact of various factors, including odd viscosity, thermocapillary effects, and bottom steepness. By taking these factors into account, we derived the non-linear evolution equation. Temporal linear stability analysis are performed based on Equation (55), and we find that the odd viscosity has a stabilizing effect, while the Marangoni number always has a destabilizing effect. We observed that regardless of the presence of odd viscosity or the value of the Marangoni number, the fluid flow is consistently more stable at the “uphill” point compared to the “downhill” point. This distinction is a consequence of different signs of the local inclination angle.

In the investigation of weakly non-linear stability, we employed the multiple scales method to derive the Ginzburg–Landau equation. By analyzing the signs of $\omega_{i}$ and $J_{2}$, we have identified four distinct nonlinear regions. We find that the subcritical instability region II and the unconditional stability region III enlarge with odd viscosity μ increases, and conversely, the supercritical stability region I and the explosive region IV shrink. While the results for the Marangoni effect are opposite to the odd viscosity effect.

To gain a deeper understanding of the stability of liquid film flow and investigate the influence of different parameters, we employ the fast Fourier transform method to solve the non-linear Equation (55). The results obtained from the numerical simulations are in agreement with the findings of the previous linear stability analysis. Numerical simulations indicate that, increasing odd viscosity diminishes perturbations and decreases the wave’s maximum height, which is precisely the opposite to the Marangoni effect. While with the increase in the bottom steepness, the height of the wave amplitude lowers first and then rises.

## Data Availability

The data that support the findings of this study are available from the corresponding author upon reasonable request.
